# Use of *in vitro* tests to assess the causative drugs for NSAIDs-induced type I hypersensitivity

**DOI:** 10.1186/2045-7022-4-S3-P31

**Published:** 2014-07-18

**Authors:** Wan-Chun Chang, Bi-Kai Hsu, Wen-Hung Chung, Shuen-Iu Hung

**Affiliations:** 1 National Yang-Ming University, Institution of Pharmacology, Taiwan; 2 Chang Gung Memorial Hospital, Department of Dermatology, Taiwan

## 

Non-steroidal anti-inflammatory drugs (NSAIDs) are associated with type I hypersensitivity, including cutaneous manifestations (e.g., urticaria and angioedema), respiratory manifestations (rhinitis, nasal polyposis, asthma), and anaphylaxis. To assess the sensitivity/specificity of *in vitro* tests for verifying the causative/tolerant drugs and cross-reactivity of different NSAIDs, we used three assays, including two ELISA tests for histamine/leukotriene C4 (LTC4) release, and a flow cytometric basophil activation test (BAT). We recruited 82 patients with NSAIDs-induced type I hypersensitivity (angioedema of the most cases), and isolated the peripheral leukocytes to perform histamine/LTC4 release tests in 38 patients, and BAT in 44 patients. The cell response to NSAIDs was examined in the incubation containing suspected or tolerant drugs with concentrations equal to 1-fold (physical level) or 10-fold Cmax. Comparing with the data of solve controls of the same subject, positive response was considered if the histamine/LTC4 release showed 1.2-fold increase, and BAT detected more than 5% increase of CD63+/CCR3+ cells. We found that histamine release test had a sensitivity of 41.9% (26/62), and specificity of 100% (10/10).LTC4 release test had a sensitivity of 32.3% (20/62), and specificity of 90% (9/10). BAT had a sensitivity of 46.4% (39/84) and specificity of 90% (18/20) for NSAIDs-induced type I hypersensitivity. Regarding drug classification, the four most common clinically suspected agents were aspirin, ibuprofen, diclofenac, and acetaminophen. The three assays have consistently >50% sensitivity for verifying aspirin, but various sensitivity and diverse individual response for the other NSAIDs. Heterogeneous mechanisms including both immunology and pharmacology may be involved in NSAIDs-induced type I hypersensitivity.

